# Editorial: Model organisms in predictive toxicology 2022

**DOI:** 10.3389/fphar.2023.1205945

**Published:** 2023-05-02

**Authors:** Yuhei Nishimura, Tetsuhiro Kudoh, Munekazu Komada

**Affiliations:** ^1^ Department of Integrative Pharmacology, Mie University Graduate School of Medicine, Tsu, Mie, Japan; ^2^ Department of Biosciences, University of Exeter, Exeter, United Kingdom; ^3^ Mammalian Embryology, Department of Life Science, Faculty of Science and Engineering, Kindai University, Higashiosaka, Osaka, Japan

**Keywords:** *in vivo* modeling, phenotype, adverse outcome pathways, context-dependency, species difference

Various chemicals, including pharmaceuticals, pesticides, microparticles, and heavy metals, are persistent in the environment (Brack et al., 2022). Predicting the toxicities of these chemicals is critical for human health and assessment of environmental risk (Michelangeli et al., 2022; Pognan et al., 2023). Next-generation technologies have developed novel *in silico* and *in vitro* approaches for risk assessments (Cavasotto and Scardino, 2022; Jeong et al., 2022). However, *in vivo* phenotypic assessments using model organisms, including fishes, rodents, and monkeys, are still indispensable for predicting toxicology and elucidating adverse outcome pathways (Komada et al., 2017; Donovan et al., 2018; Alsakran and Kudoh, 2021; Hoffmann et al., 2021; Khabib et al., 2022; Nishimura and Kurosawa, 2022).

Wang et al. demonstrated that ibrutinib, a clinical drug used in patients with B-cell malignancies, caused impairment of vascular development in zebrafish larvae. It reduced the proliferation and increased the apoptosis of vascular endothelial cells in zebrafish larvae. Additionally, ibrutinib decreased the expression of vascular endothelial cell growth factor receptors, which may have a possible role in the impairment of vascular development. Although ibrutinib has been used as a selective inhibitor of Bruton’s kinase (BTK), its toxicological effect on vascular development may not be mediated via the inhibition of BTK, since vascular development is not impaired by other BTK inhibitors or knockdown of BTK in zebrafish. Furthermore, zebrafish has also been used to develop selective BTK inhibitors (Sousa et al., 2022).

Komoike et al. showed that exposure of zebrafish embryos from 6 to 72 h post fertilization (hpf) stage to lead (Pb) at 100 ppb, which is below the occupational regulatory standard concentrations, increased the expression of oxidative stress related genes at 72 hpf. This resulted in edema and inflation defects in the swim bladder at 7 days post fertilization. These results corroborated with those of previous studies (Park et al., 2020; Wang et al., 2022), thereby, suggesting that zebrafish are useful for investigating the adverse developmental effects of trace pollutants such as Pb. As Pb easily binds to oxygen and sulfur atoms in proteins to form a stable complex and accumulates in the zebrafish body, the developmental effects of Pb at concentrations lower than 100 ppm need to be analyzed.

Using rainbow trout, Mallik et al. performed a pharmacokinetic analysis and biosafety evaluation of florfenicol, a synthetic veterinary antimicrobial agent approved by the Food and Agriculture Organization. Florfenicol has been widely used in veterinary medicine to treat and prevent diseases, and residues in food from animals treated with florfenicol can adversely affect human health (Guidi et al., 2017). In Atlantic salmon aquaculture, florfenicol is typically administered at 10 mg/kg body weight for 10 consecutive days (Horsberg et al., 1996). Mallik et al. analyzed the pharmacokinetics and biosafety of rainbow trout treated with various doses of florfenicol and recommended the use of 10 mg/kg body weight. Further *in vivo* studies are required to determine the dose of veterinary antimicrobial substances that have positive effects on animals but no adverse effects on both animals and humans who consume food products from the animals.

The antioxidant system plays a critical role in the body’s response to chemical exposure (Espinosa-Diez et al., 2015). Nuclear factor erythroid 2-related factor 2 (Nrf2) is a key transcription factor that regulates antioxidant genes (Bellezza et al., 2018). Mice have been successfully used in studies to analyze the role of Nrf2 in regard to the toxicity of many chemicals (Mutter et al., 2015).

Wang et al. examined the role of Nrf2 in the nephrotoxicity of colistin, an antibiotic known as polymyxin E. Although colistin is effective against multidrug-resistant gram-negative bacteria, its nephrotoxicity limits its use (Ordooei Javan et al., 2015; Trimble et al., 2016). Using mice, Wang et al. revealed that colistin-induced nephrotoxicity via suppression of Nrf2 was mediated by the activation of histone deacetylase 1 (Hdac1). Furthermore, co-treatment with 7-hydroxycoumarin ameliorated colistin-induced nephrotoxicity via the inhibition of Hdac1 activity. However, the mechanisms by which colistin and 7-hydroxycoumarin regulate Hdac1 warrant further investigation.

Ding et al. analyzed the role of Nrf2 in lung damage in mice exposed to ambient fine particulate matter (PM2.5) and found that lung damage was ameliorated in *Nrf2* knockout mice. In the study, it was demonstrated that endoplasmic reticulum stress caused by exposure to PM2.5, was decreased in *Nrf2* knockout mice, possibly through the suppression of Cyp2e1. The role of Nrf2 in chemical exposure and disease varies depending on the context (Menegon et al., 2016; Cheryl et al., 2021). Further studies are required to elucidate the molecular mechanisms underlying the context-dependent roles of Nrf2.

Assessment of drug-induced QT interval prolongation is a critical step in drug development (Komatsu et al., 2019). Monkeys have been widely used to assess drug-induced QT interval prolongation because they are more sensitive than dogs in this assessment, and the concentration-QT relationship is transferable to humans (Holzgrefe et al., 2014; Dubois et al., 2016). Izumi-Nakaseko et al. analyzed the effect of atrioventricular block on *dl*-sotalol-induced QT interval prolongation. They revealed that monkeys with chronic atrioventricular block could be used as a proarrhythmic model to detect drug-induced QT interval prolongation, although it takes several months to complete pathological remodeling after the onset of atrioventricular block in monkeys.

The integration of *in vivo*, *in vitro*, and *in silico* models with new methodologies is fundamental for accurately predicting chemical toxicity (Knudsen et al., 2021). Considering the different physiology, body size and sensitivities to different toxicants, it is important to investigate chemical toxicities using a variety of model organisms. Therefore, the development of novel approaches to predict toxicology using model organisms warrants further investigation.

## References

[B1] Alsakran A. Kudoh T. (2021). Zebrafish as a model for fetal alcohol spectrum disorders. Front. Pharmacol. 12, 721924. English. 10.3389/fphar.2021.721924 34975467 PMC8714738

[B2] Bellezza I. Giambanco I. Minelli A. Donato R. (2018). Nrf2-Keap1 signaling in oxidative and reductive stress. Biochim. Biophys. Acta Mol. Cell Res. 1865(5), 721–733. eng. Epub 2018/03/03. 10.1016/j.bbamcr.2018.02.010 29499228

[B3] Brack W. Barcelo Culleres D. Boxall A. B. A. Budzinski H. Castiglioni S. Covaci A. (2022). One planet: One health. A call to support the initiative on a global science–policy body on chemicals and waste. Environ. Sci. Eur. 34 (1), 21. 10.1186/s12302-022-00602-6 35281760 PMC8902847

[B4] Cavasotto C. N. Scardino V. (2022). Machine learning toxicity prediction: Latest advances by toxicity end point. ACS Omega 7 (51), 47536–47546. 10.1021/acsomega.2c05693 36591139 PMC9798519

[B5] Cheryl E. R. Yining J. Allison P. B. Luca M. K. Saamera A. (2021). The complicated role of Nrf2 in allergy and asthma. Drug Metabolism Dispos. 2021, 000414. 10.1124/dmd.121.000414

[B6] Donovan K. A. An J. Nowak R. P. Yuan J. C. Fink E. C. Berry B. C. (2018). Thalidomide promotes degradation of SALL4, a transcription factor implicated in Duane Radial Ray syndrome. eLife 7, e38430. 10.7554/eLife.38430 30067223 PMC6156078

[B7] Dubois V. F. de Witte W. E. Visser S. A. Danhof M. Della Pasqua O. Cardiovascular Safety Project Team (2016). Assessment of interspecies differences in drug-induced QTc interval prolongation in cynomolgus monkeys, dogs and humans. Pharm. Res. 33(1), 40–51. eng. Epub 2015/11/11. 10.1007/s11095-015-1760-9 26553352 PMC4689776

[B8] Espinosa-Diez C. Miguel V. Mennerich D. Kietzmann T. Sánchez-Pérez P. Cadenas S. (2015). Antioxidant responses and cellular adjustments to oxidative stress. Redox Biol. 6, 183–197. eng. Epub 2015/08/04. 10.1016/j.redox.2015.07.008 26233704 PMC4534574

[B9] Guidi L. R. Tette P. A. Fernandes C. Silva L. H. Gloria M. B. (2017). Advances on the chromatographic determination of amphenicols in food. Talanta 162, 324–338. eng. Epub 2016/11/14. 10.1016/j.talanta.2016.09.068 27837837

[B10] Hoffmann S. Marigliani B. Akgün-Ölmez S. G. Ireland D. Cruz R. Busquet F. (2021). A systematic review to compare chemical hazard predictions of the zebrafish embryotoxicity test with mammalian prenatal developmental toxicity. Toxicol. Sci. 183 (1), 14–35. eng. Epub 2021/06/11. 10.1093/toxsci/kfab072 34109416 PMC8404989

[B11] Holzgrefe H. Ferber G. Champeroux P. Gill M. Honda M. Greiter-Wilke A. (2014). Preclinical QT safety assessment: Cross-species comparisons and human translation from an industry consortium. J. Pharmacol. Toxicol. Methods 69(1), 61–101. eng. Epub 2013/05/22. 10.1016/j.vascn.2013.05.004 23689033

[B12] Horsberg T. E. Hoff K. A. Nordmo R. (1996). Pharmacokinetics of florfenicol and its metabolite florfenicol amine in atlantic salmon. J. Aquatic Animal Health 8 (4), 292–301. 10.1577/1548-8667(1996)008<0292:POFAIM>2.3.CO;2

[B13] Jeong J. Kim D. Choi J. (2022). Application of ToxCast/Tox21 data for toxicity mechanism-based evaluation and prioritization of environmental chemicals: Perspective and limitations. Toxicol. vitro Int. J. Publ. Assoc. BIBRA 84, 105451. 10.1016/j.tiv.2022.105451 35921976

[B14] Khabib M. N. H. Sivasanku Y. Lee H. B. Kumar S. Kue C. S. (2022). Alternative animal models in predictive toxicology. Toxicology 465, 153053. 10.1016/j.tox.2021.153053 34838596

[B15] Knudsen T. B. Fitzpatrick S. C. De Abrew K. N. Birnbaum L. S. Chappelle A. Daston G. P. (2021). FutureTox IV workshop summary: Predictive toxicology for healthy children. Toxicol. Sci. 180 (2), 198–211. 10.1093/toxsci/kfab013 33555348 PMC8041457

[B16] Komada M. Hara N. Kawachi S. Kawachi K. Kagawa N. Nagao T. (2017). Mechanisms underlying neuro-inflammation and neurodevelopmental toxicity in the mouse neocortex following prenatal exposure to ethanol. Sci. Rep. 7 (1), 4934. Cited in: Pubmed; PMID 28694481. 10.1038/s41598-017-04289-1 28694481 PMC5504035

[B17] Komatsu R. Mizuno H. Ishizaka T. Ito A. Jikuzono T. Kakoi T. (2019). Exposure-response analysis of drug-induced QT interval prolongation in telemetered monkeys for translational prediction to human. J. Pharmacol. Toxicol. Methods 99, 106606. 10.1016/j.vascn.2019.106606 31255745

[B18] Menegon S. Columbano A. Giordano S. (2016). The dual roles of NRF2 in cancer. Trends Mol. Med. 22(7), 578–593. eng. Epub 2016/06/07. 10.1016/j.molmed.2016.05.00 27263465

[B19] Michelangeli M. Martin J. M. Pinter-Wollman N. Ioannou C. C. McCallum E. S. Bertram M. G. (2022). Predicting the impacts of chemical pollutants on animal groups. Trends Ecol. Evol. 37 (9), 789–802. 10.1016/j.tree.2022.05.009 35718586

[B20] Mutter F. E. Park B. K. Copple I. M. (2015). Value of monitoring Nrf2 activity for the detection of chemical and oxidative stress. Biochem. Soc. Trans. 43 (4), 657–662. eng. Epub 2015/11/10. 10.1042/bst20150044 26551708 PMC4613517

[B21] Nishimura Y. Kurosawa K. (2022). Analysis of gene-environment interactions related to developmental disorders. Front. Pharmacol. 13, 863664. 10.3389/fphar.2022.863664 35370658 PMC8969575

[B22] Ordooei Javan A. Shokouhi S. Sahraei Z. (2015). A review on colistin nephrotoxicity. Eur. J. Clin. Pharmacol. 71(7), 801–810. eng. Epub 2015/05/27. 10.1007/s00228-015-1865-4 26008213

[B23] Park K. Han E. J. Ahn G. Kwak I-S. (2020). Effects of thermal stress-induced lead (Pb) toxicity on apoptotic cell death, inflammatory response, oxidative defense, and DNA methylation in zebrafish (*Danio rerio*) embryos. Aquat. Toxicol. 224, 105479. 10.1016/j.aquatox.2020.105479 32417751

[B24] Pognan F. Beilmann M. Boonen H. C. M. Czich A. Dear G. Hewitt P. (2023). The evolving role of investigative toxicology in the pharmaceutical industry. Nat. Rev. Drug Discov. 22, 317–335. 10.1038/s41573-022-00633-x 36781957 PMC9924869

[B25] Sousa B. B. de Almeida C. R. Barahona A. F. Lopes R. Martins-Logrado A. Cavaco M. (2022). Selective inhibition of Bruton's tyrosine kinase by a designed covalent ligand leads to potent therapeutic efficacy in blood cancers relative to clinically used inhibitors. ACS Pharmacol. Transl. Sci. 5 (11), 1156–1168. eng. Epub 2022/11/22. 10.1021/acsptsci.2c00163 36407952 PMC9667546

[B26] Trimble M. J. Mlynárčik P. Kolář M. Hancock R. E. (2016). Polymyxin: Alternative mechanisms of action and resistance. Cold Spring Harb. Perspect. Med. 6, a025288. 10.1101/cshperspect.a025288 27503996 PMC5046685

[B27] Wang Z. Zhao H. Xu Y. Zhao J. Song Z. Bi Y. (2022). Early-life lead exposure induces long-term toxicity in the central nervous system: From zebrafish larvae to juveniles and adults. Sci. Total Environ. 804, 150185. 10.1016/j.scitotenv.2021.150185 34509844

